# Neoplasms arising at the CIED pocket: a hybrid study combining a case report, scoping review, and clinical survey

**DOI:** 10.1007/s11739-025-04251-4

**Published:** 2026-01-08

**Authors:** Francesca Fortunato, Ciro Iardino, Veronica Abate, Maria D’Armiento, Antonio Rapacciuolo, Daniele Faccenda, Alfonso Varriale, Pietro Venetucci, Antonio Barbato, Silvana De Bonis, Giovanni Bisignani, Vincenzo Damiano, Anita Vergatti, Gianpaolo De Filippo, Domenico Rendina

**Affiliations:** 1 Department of Clinical and Experimental Medicine, Federico II University, Federico II, Via S. Pansini 5, 80131 Naples, Italy; 2 Department of Public Health, Federico II University, Naples, Italy; 3 Department of Advanced Biomedical Sciences, Federico II University of Naples, Naples, Italy; 4 Radiology Unit, Federico II University Hospital, Naples, Italy; 5 Cardiology Department, Giannetasio Hospital, ASP Cosenza, 87064 Rossano, Italy; 6 Cardiology Department, Ferrari Hospital, ASP Cosenza, 87012 Castrovillari, Italy; 7 Assistance Publique-Hôpitaux de Paris, Hôpital Robert Debré, Service d’Endocrinologie Et Diabétologie, 75019 Paris, France

**Keywords:** CIED, Cancer, Neoplasm, Metastasis

## Abstract

Cardiac implantable electronic device (CIED) implantation is a minor surgical procedure, with a low complication incidence. Neoplasm occurrence at the CIED pocket is a rare event, and sporadic cases are reported without a comprehensive evaluation. This hybrid study aims to fill this gap through a three-part approach: a new case report, a scoping review with individual patient data (IPD) analysis, and a clinical survey. In March 2024, a male patient with a history of high-grade urothelial cancer referred to our Department with a mass at the CIED pocket. Incisional biopsy confirmed the diagnosis of high-grade urothelial carcinoma. The patient died after 4 months. Through our scoping review and IPD analysis, we collected 56 patients affected by primary neoplasms [40 (71.4%)] and metastasis [16 (28.6%)] at the CIED pocket. Among the primary neoplasms, adenocarcinoma (12; 30.0%) was most frequent in females, while sarcoma (10; 25.0%) was predominant in males. On the other hand, metastasis types were represented by breast cancer (3; 18.8%) in females, and myeloma (2; 13.3%) and melanoma (2; 13.3%) in males. The clinical survey showed data from 471 patients who underwent CIED implantation. No primary neoplasm or metastasis developed at the CIED pocket, also in subjects with history of cancer. Although rare, both primary and metastatic neoplasms may occur at the site of CIED implantation. Vigilant monitoring of CIED patients is essential, particularly when changes at the pocket site arise. Early biopsy and oncologic assessment should be considered in such cases to enable timely diagnosis and intervention.

**Supplementary Information:**

The online version contains supplementary material available at 10.1007/s11739-025-04251-4.

## Introduction

Since their introduction in the 1960s, cardiac implantable electronic devices (CIEDs), including permanent pacemakers and implantable cardioverter-defibrillators, have represented a significant breakthrough in the clinical management of patients with life-threatening cardiac arrhythmias [1]. Originally developed as large external devices, technological advancements have led to the evolution of implantable CIEDs. These are inserted into a subcutaneous pocket and connected to cardiac tissue via one or more leads (single- or dual-chambers systems). More recently, leadless CIEDs that are directly anchored to the ventricular wall have been introduced [2, 3]. According to the 2021 cardiac pacing guidelines CIED implantation is indicated for patients with sinus node dysfunction, advanced or paroxysmal atrioventricular block, conduction disorder without atrioventricular block, and reflex syncope [4]. Approximately, 1.25 million CIEDs are implanted each year, although implantation rates vary by economic, socio-cultural, health and geographical factors [3]. CIED implantation is considered a minor surgery with a relatively low incidence of complications [5]. The most common complications, each occurring in fewer than 6% of cases, include cardiac tamponade, cardiac perforation, venous thrombosis, left ventricular leads alteration, leads dislodgment, local hematoma, pocket infection, and pneumothorax [3]. Neoplasia arising at the CIED pocket is an ultra-rare complication only sporadically reported and lacking systematic evaluation in the literature [6]. Therefore, its clinical characteristics, management strategies, and prognosis remain poorly defined. To fill this gap, this hybrid article presents 1) a new case of neoplasm arising at the CIED pocket, 2) a scoping review with individual patient data analysis (IPD) to define the clinical characteristics, treatment and outcomes of neoplasm arising at the CIED pocket, 3) a clinical survey conducted across 3 CIED Centers in Campania and Calabria, Southern Italy. By integrating these three perspectives, we have constructed a completer and more nuanced picture of the condition. Our hybrid design provides a more complete and nuanced understanding of this ultra-rare condition. This approach strengthens the study by combining internal validity derived from the clinical case and regional survey with the external breadth offered by worldwide evidence, a level of insight that would not have been achievable through any single methodology alone.

## Methods.

**1) Case report**. The patient has voluntarily signed his informed consent to the publication of the case report.

## 2) Scoping review with IPD analysis.

**Data sources and search strategy.** The review was planned, conducted, and reported according to the Preferred Reporting Items for Systematic reviews and Meta-Analyses (PRISMA) statement [7] (Supplementary Fig. 1). Systematic search was performed in Medline, Google Scholar, Google book, and Cochrane Library (last conducted search April 20, 2024) using the following term: “cancer AND cardiac implantable electronic devices”, "cancer pacemaker", “neoplasm leads”, “cancer pocket”, “pacemaker metastasis”, “cancer CIED”. No language restrictions were applied. The reference lists of all identified articles were searched for additional relevant publications. **Study selection.** Eligible studies included case reports and case series. All articles were obtained in full text and the references were analyzed to exclude duplicate data. The predetermined inclusion criterion was cancer arising at the CIED pocket. Predetermined exclusion criterion was cancer not CIED pocket related. **Data extraction.** Titles and abstracts (when available) of the studies retrieved using the described search strategy were screened independently by 2 review team members (C.I. and F.F.) to identify studies that potentially met the inclusion criteria outlined. The full text of potentially eligible studies was retrieved and independently assessed for eligibility by 3 review team members (D.R., A.V., V.A.). Studies in languages other than English, French, and Italian (i.e., Chinese, Spanish, Portuguese, German, and Japanese) were translated into English or Italian by a professional translator. Any disagreement over the eligibility of studies was resolved through discussion among all review team members. A standardized, pre-piloted form was used to extract relevant clinical data from the included studies. The extracted data included: sex, current age, age at CIED implantation, history of CIED replacement, age at diagnosis of neoplasm arising at the CIED pocket, histological diagnosis of CIED pocket neoplasm, CIED type, CIED components, CIED removal, neoplasm treatment, follow-up, death, age of death, causes of death, comorbidities including occurrence of neoplasm not-CIED pocket related. Two review team members (C.I. and F.F.) extracted data independently, and any discrepancies were resolved through discussion with D.R. When available, missing data were obtained upon request from study authors via mail. The critical appraisal of case report and case series included in the systematic review was conducted according to the Joanna Briggs Institute checklist for case series (Supplementary Table 1) and for case reports (Supplementary Table 2) [8].

**3) Clinical survey.** From January 1st to December 31st, 2023, all consecutive patients referred to the Department of Cardiology at the University of Naples, Castrovillari Hospital (Castrovillari, Cosenza, Italy), and “Nicola Giannattasio” Hospital (Rossano, Cosenza, Italy) for CIED implantation, were enrolled in our clinical survey. A standardized pre-piloted form was used to extract relevant data from enrolled patients’ medical records. The extracted data included: sex, current age, age at CIED implantation, diseases leading to CIED implantation, history of CIED replacement, occurrence of neoplasm arising at the CIED pocket, histological diagnosis of CIED pocket neoplasm, CIED type, CIED components, CIED removal, follow-up, death, age of death, causes of death, comorbidities including occurrence of neoplasm not-CIED pocket related. Two review team members (F.F. and C.I.) extracted data independently, and any discrepancies were resolved through discussion with D.R. Written informed consent for clinical data collection and anonymized analysis was obtained from all patients, in accordance with institutional regulations and the principles of the Declaration of Helsinki.

**Statistical analysis.** All statistical Analysis were performed using the IBM SPSS (Statistical Package for Social Science), version 25 (IBM, Armonk, NY, USA). Data from study publications were extracted and included in a single database. These data were then reanalyzed and combined. The χ2 or Fisher exact test was used to evaluate differences between categorical variables or proportions. The Kolmogorov–Smirnov test was used to assess the data distribution. Data are expressed as media ± standard deviation and [confidence interval (CI) 95%] for continuous variables with normal distribution, as median [Interquartile Range (IQR)] for continuous variables with not-normal distribution, and as absolute number (percentages) for categorical variables [9]. All statistical tests were 2-enrolltailed. A P value < 0.05 was considered statistically significant.

## Result

**Fig. 1:**
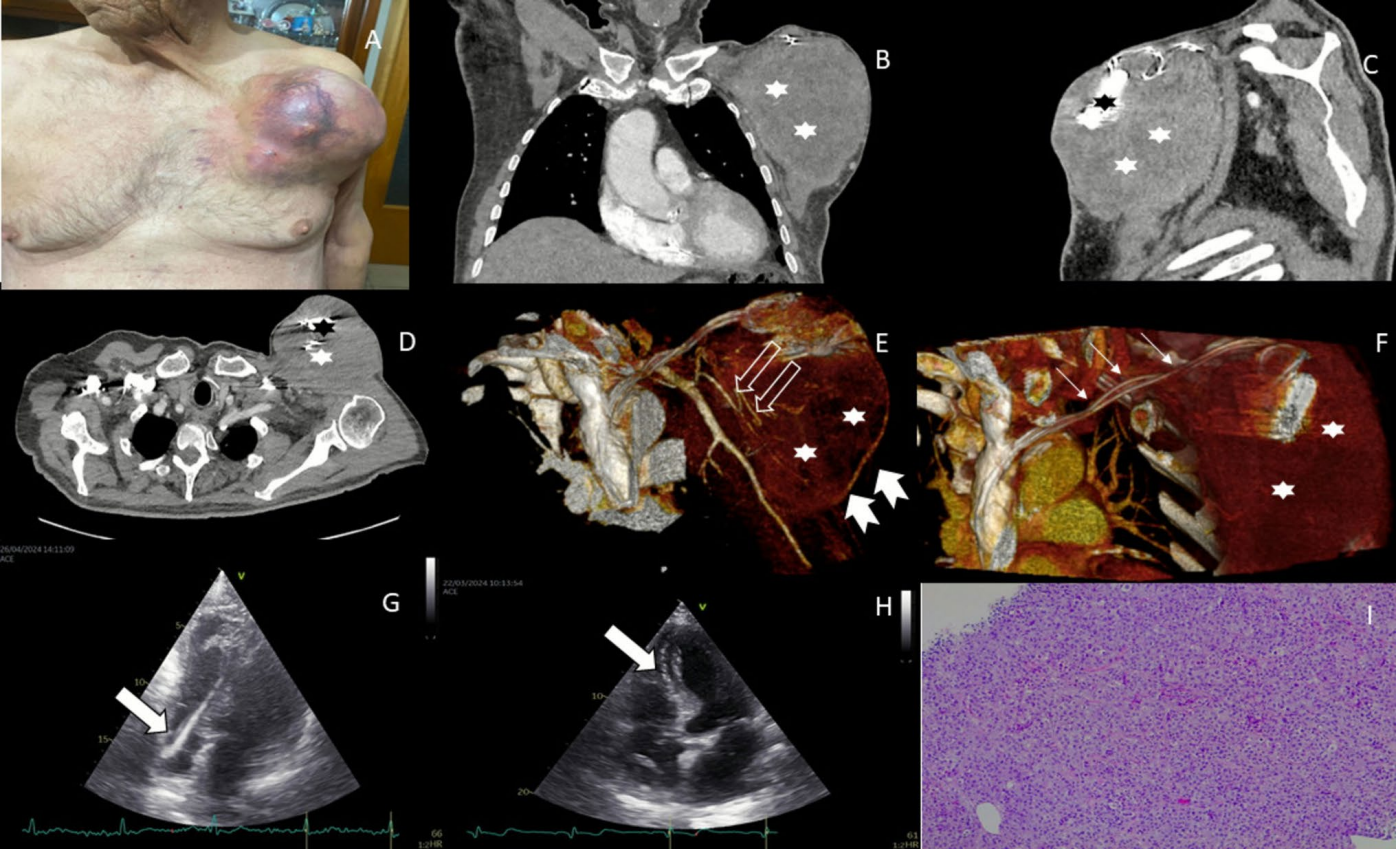
**A** Neoplasm arising at the CIED pocket. **B** Computed tomography images. **C** Coronal section. **D** transversal section. E: three-dimensional rotational angiography. **F** Three- dimensional computed tomography reconstruction. White star: device pocket neoplasm. Black star: ICD device. White arrow: device’s leads. Empty arrow: arterial circulation of the neoplasm. Arrowheads: venous circulation of the neoplasm. **G**, **H** Cardiac ultrasound examination. Apical four chamber view. White arrow: images of the vegetation on catheter. **I**. Hematoxylin–Eosin 10x; Device pocket neoplasm biopsy. Both samplings show high-grade neoplasm with diffuse and organoid patterns, medium-sized cells, with large atypical nuclei, pleomorphs

**Case report.** In March 2024, an 84-year-old Caucasian man (LM) was referred to the Department of Clinical Medicine and Surgery of the Federico II University in Naples, Italy, due to mass in CIED pocket, located on the left side of his chest. About a year earlier, he had been diagnosed with high-grade urothelial cancer, which infiltrated the muscle causing metastases (stage IV-Metastatic cancer, T1-T4, N1-N3, M1) [10, 11]. Following syncopal episodes, a double chamber CIED was implanted in February 2023. On physical examination, a mass measuring 13 cm was noted at the skin level. The mass was red, firm, warm and tender to palpation (Fig. 1 A). At the time of hospital admission, the patient’s vital signs were stable: body temperature 36,5 °C, blood pressure 135/80 mmHg, heart rate 65 bpm, and respiratory rate 15 breaths per minute. A computerized tomography (CT) scan revealed an oval mass with regular margins, measuring 10 cm in the axial plane and 15 cm in the cranial-caudal direction. The mass showed modest vascularization without signs of active bleeding, and was not separated from the underlying muscle, while the bone remained intact (Fig. 1 B-C-D-E–F). During the hospitalization, two cardiac echocardiograms were performed, revealing a jagged and mobile image in the electro-catheter at the apex of the right ventricle (2.6 × 1.6 cm). This image was difficult to distinguish from the top of the catheter (Fig. 1 G-H). Initially, vegetations were suspected, suggesting an infection. However, laboratory tests (C-reative protein, procalcitonin, leucocytes) did not indicate any infectious process, which was further confirmed by culture results. Consequently, the mass underwent incisional biopsy. Histological examination shows a high-grade cancer with a diffuse, organoid pattern composed of medium-sized cells with large atypical nuclei, pleomorphism, and central macronucleolus (Fig. 1 I). Immunohistochemical analysis of the tumor showed positivity for vimentin, cytokeratin 7, cytokeratin 8 and 18, epithelial membrane antigen, integrase Interactor 1, and beta membrane catenin. Negativity was observed for cytokeratin 5 and 6, GATA3 binding protein, chromogranin, S100, and Cd99. Histological examination of the CIED pocket revealed findings consistent with urothelial carcinoma, negative for chromogranin, synaptophysin, and anticardiolipin, but positive for pancytokeratin (Fig. 1 I). The CIED pocket mass was diagnosed as a metastasis of bladder carcinoma. Over time, the mass enlarged, ulcerated and began to bleed. He was evaluated for potential surgical removal of the mass, with possible transposition of the latissimus dorsi muscle flap. However, due to the high surgical risk, the patient was deemed inoperable and subsequently received palliative radiotherapy (5 Gy for 5 total sessions), following device deactivation. The patient died 4 months after the initial diagnosis.

**Scoping review with IPD analysis.** As showed in the Supplementary Fig. 1, 50 studies were included in qualitative and quantitative syntheses. The case reports and the case series included in the final analysis were 45 and 5, respectively. A complete list of studies included in the systematic review with IPD analysis and their critical appraisals are reported in Supplementary Tables 1 and 2. We collected data from 56 [male: female (M: F) = 37 (66.1%): 19 (33.9%), mean age 75.7 ± 13.7 years (CI 95% 72.0- 79.3)] subjects with neoplasm at CIED pocket site, including our case report. The mean age at CIED implantation was 67.9 ± 16.3 years [CI 95% 63.2–72.6] years. Sixteen subjects had a previous history of cancer [M: F = 10 (62.5%): 6 (37.5%); mean age at cancer onset 73.9 ± 19.1 (CI 95% 59.27 – 88.51) years], and 18 [M: F = 12 (66.7%): 6 (33.3%); mean age 71.6 ± 18.4 (CI 95% 62.4 – 80.7) years] underwent CIED replacement pre-cancer onset. Based on histological diagnosis, neoplasm arising at the CIED pocket are classified as primary neoplasm or metastasis (Table 1 and 2, respectively).

**Table 1:** Characteristics of subjects with primary neoplasm on PMK pocket

	N° of subjects with available data	Men (27; 66.5)	Women (13;32.5)	p-value
Age at cancer onset on CIED pocket (years)	40	77.2 [71.8–82.6]	76.7 [70.9- 82.5]	0.97
Age at CIED implantation (years)	36	69.4 [62.1- 76.7]	68.3.0 [60.9- 75.6]	0.89
CIED replacement pre-cancer	10	7 (70.0)	3 (30.0)	0.59
CIED replacement post-cancer	14	10 (71.4)	4 (28.6)	0.35
Primary neoplasm	40			
Carcinoma [1]		9 (22.5)	13 (32.5)	
Adenocarcinoma		2 (5.1)	12(30.0)	
Cutaneous squamous-cell carcinoma		4 (10.0)	1 (2.5)	
Basal-cell carcinoma		3 (7.5)	-	< 0.05
Sarcoma		10 (25.0)	-	
Hematological malignancy		8 (20.0)	-	
Therapeutic approach	36			0.03
Surgery		18 (50.0)	1 (2.8)	
CHT		2 (5.6)	3 (8.3)	
RT		-	1 (2.8)	
Surgery + CHT		2 (5.6)	3 (8.3)	
Surgery + RT		1 (2.8)	2 (5.6)	
Surgery + CHT + RT		1 (2.8)	1 (2.8)	
Follow-up (months)	23	5 [2.2–12.0]	7.0 [5.0–24.0]	0.13
Death/Alive	28	9 (32.1)/11 (39.3)	4 (14.3)/4 (14.3)	0.56

Data are expressed as mean and CI 95% for continuous variables with both normal distribution at Kolmogorov–Smirnov test and as median [25°-75°] for continuous variables with not-normal distribution at Kolmogorov–Smirnov test, and absolute number (percentage). *CIED* implantable management device, *CHT*: chemotherapy, *RT* radiotherapy

[1] https://training.seer.cancer.gov/disease/categories/classification.html (last accessed on June 21st, 2025)

**Table 2:** Characteristics of subjects with metastases on PMK pocket

	N° of subjects with available data	Men (10; 62.5)	Women (6; 37.5)	p-value
Age at metastasis onset on CIED pocket (years)	16	68.5 [56.6–80.4]	78.5 [65.9–91.0]	0.26
Age at CIED implantation (years)	12	59.0 [38.1–79.9]	71.8 [58.2–85.4]	0.27
CIED replacement pre-metastasis	8	5 (62.5)	3 (37.5)	0.41
CIED replacement post-metastasis	7	5 (71.4)	2 (28.6)	0.64
Metastasis	15			
Breast cancer		-	3 (18.8)	
Sarcoma		1 (6.7)	-	
Basal-cell carcinoma		1 (6.7)	-	
Myeloma		2 (13.3)	2 (13.3)	0.01
Lymphoma		1 (6.7)	1 (6.7)	
Melanoma		2 (13.3)	-	
Prostate cancer		1 (6.7)	-	
Urothelial cancer		1 (6.7)	-	
Therapeutic approach	15			0.89
Surgery		5 (33.3)	2 (13.3)	
CHT		2 (13.3)	1 (6.7)	
Surgery + CHT		1 (6.7)	1 (6.7)	
CHT + RT		1 (6.7)	-	
Surgery + CHT + RT		1 (6.7)	1 (6.7)	
Follow-up (months)	7	7.5 [1.0–24.0]	6.0 [-]	0.40
Death/Alive	11	3 (27.3)/5 (45.5)	3 (27.3)/-	0.12

Data are expressed as media and CI 95% for continuous variables with normal distribution at Kolmogorov–Smirnov test and median [25°-75°] for continuous variables with not-normal distribution at Kolmogorov–Smirnov test, and absolute number (percentage). *CIED* implantable management device, *CHT* chemotherapy, *RT* radiotherapy

As showed in Table 1, primary neoplasms affected 27 (66.5%) males and 13 (32.5%) females. The distribution of different cancer types between males and female was significantly different. All females were affected by adenocarcinoma, whereas males were mainly affected by sarcoma (10; 37.0%) and haematological malignancies (8; 29.6%). The onset of primary neoplasms at the CIED pocket occurs approximately 8 years after the CIED implantation, and after neoplasm diagnosis, 15 (71.4%) males and 6 (28.6%) females underwent CIED replacement. The median follow-up was 6.0 [IQR 3.0–12.0] months and 13 [M: F = 9 (69.2%): 4 (30.8)] died during the follow-up.

As showed in Table 2, metastases were observed in 10 (62.5%) males and 6 (37.5%) females. The distribution of different metastases between males and female was significantly different. Breast cancer metastasis affected typically females (3; 18.8%), whereas males were mainly affected by myeloma (2; 13.3%) and melanoma (2; 13.3%). The onset of metastasis at the CIED pocket occurs approximately 9 years after the CIED implantation, and after metastasis occurrence, 5 (71.4%) males and 2 (28.6%) females underwent CIED replacement. The median follow-up was 6.0 [IQR 2.0–18.0] months and 6 [M: F = 3(50.0%): 3 (50.0%)] died during the follow-up.

**Clinical survey.** We collected data from 471 [M: F = 339 (71.9%): 102 (28.1%); mean age 70.3 ± 9.8 years] patients who underwent CIED implantation. Forty-nine subjects [10.4%; F:M = 22 (43.0%): 27; (57.0%); mean age at CIED implantation 72.1 ± 10.3 years] had a history of cancer at CIED implantation. The most common cancer types were adenocarcinoma, followed by hematological malignancy, melanoma, and sarcoma. During a mean follow-up of 19.1 ± 7.4 months, 24 (5.0%) patients [M: F = 13 (53.4%): 11 (46.6%)] developed complications related to CIED implantation: 11 [45.8%; 3 men] pocket hematomas; 6 [25.0%; 3 men] pneumothoraxes; 6 [25.0%; 1 man] perforation tamponades; 1 (4.2%) hemothorax. No patient has developed neoplasm at the CIED pocket. The occurrence of complications was the same in subjects with and without history of cancer.

## Discussion

The results of our hybrid study highlight that, although rare, primary neoplasms and metastases may represent a potential complication of CIED implantation. These cancers are more commonly observed in the elderly population, with adenocarcinoma being the most frequent type in females, and sarcoma and hematological malignancies more common in males.

CIED implantation is generally a safe procedure, however, it carries some potential risks and complication both in the short and long term. Common complications include arrhythmias, infections, bleeding and hematomas, lead dislodgement or fracture, and venous thrombosis [12]. Despite the high number of CIED implantations performed each year, cases of neoplasm at the CIED pocket site remain rare in the current literature.

CIED can be considered a foreign body and may trigger immune responses and potentially lead to inflammation and tissue changes [13]. Chronic inflammation is known to be linked to tumorigenesis by promoting cellular transformation, proliferation, angiogenesis, and metastasis [14]. The majority of cancers are associated with environmental factors, such as chronic infections, tobacco smoke, pollution, and lifestyle [15]. All of these contribute to chronic inflammation, that is considered a main driver of tumorigenesis. The connection between inflammation and cancer can be summarized in two main pathways: an extrinsic pathway, in which inflammation increases the risk of cancer, and an intrinsic pathway, in which genetic mutations initiate inflammation and tumor development [16]. Inflammatory mediators and cells are a part of the microenvironment of cancer. Moreover, some oncogene mutations can promote inflammation and consequently cancer progression [16]. Mechanisms involved in cancer-related inflammation also play a role in invasion and metastasis. Chemokines such as interleukin-8 (IL-8) facilitate the migration of cancer cells to distant sites [17]. Inflammatory cytokines, including tumor necrosis factor-α (TNF- α), IL-1 β, and IL-6, enhance the invasive potential of cancer cells [18–20]. A key regulator in this process is NF-κB, a coordinator of innate immunity and inflammatory. NF-κB acts as an endogenous promoter of cancer and metastatic by activating genes encoding inflammatory and angiogenic factors, promoting cell survival and proliferation, and increasing the cell migration and invasion [21].

Another consequence of the persistent presence of a foreign body, such as CIED, is the overproduction of growth and angiogenic factors. These factors are involved into several stages of cancer development and progression. Growth factors can stabilize oncogenic mutation and increase the population of cells susceptible to further mutations [22]. They also play a critical role in invasion and migration of cancer cells [23], for instance by inducing the loss of epithelial E-cadherin or the acquisition of N-cadherin [24]. Vascular endothelial growth factors (VEGFs), Fibroblast-growth Factors (FGFs), and Transforming growth factor-β (TGF- β) are among the main growth factors implicated in cancer initiation, progression and metastasis [25].

As our hybrid study shows, cancers developing at the CIED pocket site are a rare occurrence, and current evidence is insufficient to fully explain this phenomenon. Indeed, although several mechanisms have been proposed to explain cancer development in proximity to CIED, these remain hypothetical in this context. Evidence from the biomaterial and oncology fields suggests that chronic inflammation and foreign body reaction may promote cellular proliferation, angiogenesis, and genomic instability, potentially facilitating tumorigenesis in susceptible individuals. However, no studies to date have demonstrated a direct causal relationship between CIED implantation and neoplastic transformation, and the mechanisms described should therefore be interpreted as general biological concepts rather than CIED-specific evidence. Further research is required to determine whether the biological pathways known to operate in chronic inflammatory settings also play a role in neoplasia arising at the CIED pocket.

Nevertheless, patients with CIED should be routinely monitored, and any changes in the pocket site should prompt cancer screening and appropriate treatment. CIED remains a life-saving device, especially in cancer patients by increasing their chances of undergoing treatment and improving survival outcomes.

Strengths and limitations of this study are represented by its systematic review nature. Unlike the systematic review by Moraes et al. [6], which focused exclusively on pacemaker-associated neoplasms and included only published cases, our study encompasses all types of CIEDs and integrates literature data with an original clinical cohort, providing a broader, more contemporary perspective and a real-world clinical experience. Indeed, it enables the collection and synthesis of global data on an extremely rare clinical condition, the heterogenicity of the same cannot guarantee an even and consistent approach. Moreover, the available data is limited to what reported by the authors. Another limitation is represented by the uniqueness of the complication; therefore, a small number of cases have been collected. In addition, the absence of neoplasms in the clinical survey should be interpreted with caution, as the mean follow-up of 19 months is relatively short and may not be sufficient to detect late-onset pocket neoplasms.

This study highlights that neoplastic transformation within CIED pocket is a rare but life-threatening complication. Despite the implementation of multimodal treatment strategies—including chemotherapy, radiotherapy, and surgery—prognosis remains poor, with a marked reduction in life expectancy. Although routine screening for pocket neoplasms cannot be recommended due to their extreme rarity, clinicians should maintain a high index of suspicion when evaluating atypical pocket changes, especially in patients presenting with persistent inflammation, rapidly growing masses, ulceration, or unusual pain. Early targeted imaging (ultrasound as first-line, followed by CT or MRI when deeper extension is suspected) may help characterize the lesion and guide further management. When clinical or imaging findings raise concern for malignancy, timely biopsy is recommended to avoid diagnostic delays. At present, no evidence supports formal surveillance protocols, but careful clinical follow-up after CIED interventions and prompt investigation of non-resolving pocket abnormalities may facilitate earlier detection of rare neoplastic transformations.

## Supplementary Information

Below is the link to the electronic supplementary material.

Supplementary file1 (DOCX 32 KB)

Supplementary file2 (DOCX 18 KB)

Supplementary file3 (TIF 96 KB)

## Data Availability

The datasets used and/or analysed during the current study are available from the corresponding author on reasonable request.
