# Supplementary material for: Neoplasms arising at the CIED pocket: a hybrid study combining a case report, scoping review, and clinical survey
Source: Intern Emerg Med. 2026 Jan 8;21(3):957–64. doi: 10.1007/s11739-025-04251-4 (PMC13144183; doi:10.1007/s11739-025-04251-4)
Supplement: Supplementary file 1 — Supplementary file1 (DOCX 32 KB) [file 11739_2025_4251_MOESM1_ESM.docx]

**Supplementary Table 1. Quality assessment for case reports.**

|  | **JBI Critical Appraisal Checklist for Case Reports** | | | | | | | |
| --- | --- | --- | --- | --- | --- | --- | --- | --- |
| **Ref.** | **1** | **2** | **3** | **4** | **5** | **6** | **7** | **8** |
|  | Y | Y | Y | Y | Y | Y | Y | Y |
|  | Y | Y | Y | Y | Y | Y | Y | Y |
|  | Y | Y | Y | Y | Y | Y | Y | Y |
|  | Y | Y | Y | Y | Y | N | N | Y |
|  | Y | N | Y | Y | Y | Y | Y | Y |
|  | Y | Y | Y | Y | Y | Y | Y | Y |
|  | N | N | N | N | N | N | N | N |
|  | Y | Y | Y | Y | Y | Y | Y | Y |
|  | Y | Y | Y | Y | Y | Y | Y | Y |
|  | Y | Y | Y | Y | Y | Y | Y | Y |
|  | Y | N | Y | Y | Y | N | N | Y |
|  | Y | N | Y | Y | Y | N | N | N |
|  | Y | Y | Y | Y | Y | Y | Y | Y |
|  | Y | Y | Y | Y | Y | Y | Y | Y |
|  | Y | Y | Y | Y | Y | Y | Y | Y |
|  | Y | Y | Y | Y | Y | Y | Y | Y |
|  | Y | Y | Y | Y | Y | Y | Y | Y |
|  | Y | N | N | Y | Y | N | N | N |
|  | Y | Y | Y | Y | Y | Y | Y | Y |
|  | Y | Y | Y | Y | Y | Y | Y | Y |
|  | Y | N | Y | Y | Y | Y | Y | Y |
|  | Y | Y | Y | Y | Y | N | N | Y |
|  | Y | Y | Y | Y | Y | Y | Y | Y |
|  | Y | Y | Y | Y | Y | Y | Y | Y |
|  | Y | Y | Y | Y | Y | Y | Y | Y |
|  | Y | Y | Y | Y | Y | Y | Y | Y |
|  | Y | N | Y | Y | Y | N | N | Y |
|  | Y | Y | Y | Y | Y | Y | Y | Y |
|  | Y | Y | Y | Y | Y | Y | Y | Y |
|  | Y | N | Y | Y | Y | Y | Y | Y |
|  | Y | Y | Y | Y | Y | Y | Y | Y |
|  | Y | N | Y | Y | Y | N | N | Y |
|  | Y | N | Y | Y | Y | Y | Y | Y |
|  | Y | N | Y | Y | Y | N | N | Y |
|  | Y | N | Y | Y | Y | Y | Y | Y |
|  | Y | Y | Y | Y | Y | Y | Y | Y |
|  | Y | N | N | N | N | N | N | NA |
|  | Y | N | Y | N | N | N | N | Y |
|  | Y | Y | Y | Y | Y | Y | Y | Y |
|  | Y | N | Y | Y | Y | N | N | Y |
|  | Y | Y | Y | Y | Y | N | N | Y |
|  | Y | Y | Y | Y | Y | Y | Y | Y |
|  | Y | N | Y | Y | Y | Y | Y | Y |
|  | Y | Y | Y | Y | Y | Y | Y | Y |
|  | Y | Y | Y | Y | Y | N | N | Y |

1. Were patient’s demographic characteristics clearly described?
2. Was the patient’s history clearly described and presented as a timeline?
3. Was the current clinical condition of the patient on presentation clearly described?
4. Were diagnostic tests or assessment methods and the results clearly described?
5. Was the intervention(s) or treatment procedure(s) clearly described?
6. Was the post-intervention clinical condition clearly described?
7. Were adverse events (harms) or unanticipated events identified and described?
8. Does the case report provide takeaway lessons?

Ref: references. Yes: Y. No: N. U: Unclear. Not applicable: N.A.

Moola S, Munn Z, Tufanaru C, Aromataris E, Sears K, Sfetcu R, Currie M, Lisy K, QureYshi R, Mattis P, Mu P. Chapter 7: Systematic reviews of etiology and risk. In: Aromataris E, Munn Z (Editors)*. JBI Manual for Evidence Synthesis.* JBI, 2020. Available from https://synthesismanual.jbi.global. <https://doi.org/10.46658/JBIMES-20-08>

**References**

1. Bodagh N, Pappa E, Farooqi F. Multidisciplinary surgical team approach for excision of squamous cell carcinoma overlying pacemaker site. BMJ Case Rep. 2018 Feb 6;2018:bcr2017221660. doi: 10.1136/bcr-2017-221660. PMID: 29437678; PMCID: PMC5847853.
2. Heiko Burger, Gerhard Göbel, Manfred Richter, Simon Pecha, Case report of a large lipoma discovered intraoperatively in a chronically irritated implantable cardioverter-defibrillator pocket, European Heart Journal - Case Reports, Volume 6, Issue 7, July 2022, ytac245, https://doi.org/10.1093/ehjcr/ytac245
3. Burke EM, Brown CL, Kamenske J, Burke MC. Basal cell carcinoma overlying a pacemaker pocket in a pacemaker-dependent patient: Management and course. HeartRhythm Case Rep. 2024 Feb 2;10(4):276-279. doi: 10.1016/j.hrcr.2024.01.013. PMID: 38766608; PMCID: PMC11096418.
4. Carpentier O, Dubost-Brama A, Martin De Lassalle E, Piette F, Delaporte E. Rhabdomyosarcome sur site d'implantation d'un stimulateur cardiaque [Rhabdomyosarcoma at site of pacemaker implantation]. Ann Dermatol Venereol. 2000 Oct;127(10):837-40. French. PMID: 11060388.
5. Sia CH, Goh FQ, Kong WK, Wu B, Paranjothy S. Cardiac sarcoma attached to pacemaker lead. J Card Surg. 2020 May;35(5):1148-1151. doi: 10.1111/jocs.14541. Epub 2020 Apr 15. PMID: 32293046.
6. D'Arienzo G, Sicuranza M, Ziccardi L, Di Biase M, Brunetti ND. Leadless implantation as alternative for pacemaker replacement in infiltrative breast cancer. J Cardiovasc Med (Hagerstown). 2021 Mar 1;22(3):222-224. doi: 10.2459/JCM.0000000000001026. PMID: 33512976.
7. De Mattia L, Brieda M, Dametto E. A carcinoma of the breast mimicking a pacemaker pocket infection. Europace. 2011 Feb;13(2):220. doi: 10.1093/europace/euq369. Epub 2010 Oct 12. PMID: 20940183.
8. Magilligan DJ Jr, Isshak G. Carcinoma of the breast in a pacemaker pocket--simple recurrence or oncotaxis? Pacing Clin Electrophysiol. 1980 Mar;3(2):220-3. doi: 10.1111/j.1540-8159.1980.tb04332.x. PMID: 6160512.
9. Flecher E, Bertheuil N, Leclercq C, Verhoye JP. Cancer on a pacemaker lead. Int J Cardiol. 2011 Sep 1;151(2):e56-7. doi: 10.1016/j.ijcard.2010.04.098. Epub 2010 Jun 1. PMID: 20684852.
10. Fleißner F, Molitoris U, Rösler W, Kühn C. Primary Cardiac B-Non-Hodgkin Lymphoma Disguised as a Pacemaker Endocarditis. Thorac Cardiovasc Surg Rep. 2018 Jan;7(1):e18-e20. doi: 10.1055/s-0038-1660807. Epub 2018 Jun 28. PMID: 29977733; PMCID: PMC6023713.
11. Fraedrich G, Kracht J, Scheld HH, Jundt G, Mulch J. Sarcoma of the lung in a pacemaker pocket--simple coincidence or oncotaxis? Thorac Cardiovasc Surg. 1984 Feb;32(1):67-9. doi: 10.1055/s-2007-1023349. PMID: 6198780.
12. Golzio PG, Cristoforetti Y, Gaita F. Melanoma metastasis over a pacemaker pocket: integrated management strategies and facilitating factors. Europace. 2013 Oct;15(10):1490. doi: 10.1093/europace/eut046. Epub 2013 Mar 12. PMID: 23482615.
13. González-Vela MC, Salcedo W, Neira C, González-López MA, Ayala H, Val-Bernal JF. Atypical fibroxanthoma developing on a pacemaker pocket mimicking a pyogenic granuloma. Cardiovasc Pathol. 2013 Jan-Feb;22(1):102-4. doi: 10.1016/j.carpath.2012.03.006. Epub 2012 Apr 12. PMID: 22502867.
14. González-Vela MC, Val-Bernal JF, Rubio S, Olalla JJ, González-López MA. Cutaneous leiomyosarcoma developing on a pacemaker pocket. Dermatol Surg. 2009 May;35(5):863-7. doi: 10.1111/j.1524-4725.2009.01145.x. Epub 2009 Mar 30. PMID: 19389092.
15. Hamaker WR, Lindell ME, Gomez AC. Plasmacytoma arising in a pacemaker pocket. Ann Thorac Surg. 1976 Apr;21(4):354-6. doi: 10.1016/s0003-4975(10)64327-5. PMID: 1267518.
16. Herrmann JL, Mishra V, Greenway HT. Basal cell carcinoma overlying a cardiac pacemaker successfully treated using Mohs micrographic surgery. Dermatol Surg. 2014 Apr;40(4):474-7. doi: 10.1111/dsu.12436. Epub 2014 Jan 23. PMID: 24456290.
17. Khamooshian A, Klinkenberg TJ, Maass AH, Mariani MA. Management of device-related malignant sarcoma. HeartRhythm Case Rep. 2017 Jun 13;3(8):373-376. doi: 10.1016/j.hrcr.2017.04.002. PMID: 28840102; PMCID: PMC5558189.
18. Liao JN, Chen IM, Yang AH, Yu WC. A primary cardiac sarcoma spreading along the pacing leads of a permanent pacemaker. J Am Coll Cardiol. 2012 Apr 17;59(16):1487. doi: 10.1016/j.jacc.2011.07.062. PMID: 22497829.
19. Knez I, Cerwenka H, Moinfar F, Hoff M, Mächler H, Anelli-Monti M, Radner H, Rigler B. Invasive ductal carcinoma of the male breast expanding from pacemaker pocket decubitus. Pacing Clin Electrophysiol. 1999 Mar;22(3):531-3. doi: 10.1111/j.1540-8159.1999.tb00484.x. PMID: 10192865.
20. Li A, Chen A, Gallagher M, Kaczmarek P, Tinwell B, Sneddon J, Cliff S. Primary cutaneous plasmacytoma occurring after pacemaker implantation and recurring in scar tissue. Dermatol Online J. 2013 Feb 15;19(2):3. PMID: 23473273.
21. McCreary K, Roberts M, McKeag N. Pacemaker pocket mass: tumour within the deltopectoral groove. Europace. 2020 Oct 1;22(10):1536. doi: 10.1093/europace/euaa125. PMID: 32533182.
22. Mellert F, Schiller W, Yueruektuemen A, Preusse CJ, Welz A. A rare case of skin cancer above a subcutaneously implanted pacemaker: implications for future implants. Heart Surg Forum. 2008;11(3):E132-3. doi: 10.1532/HSF98.20071211. PMID: 18583279.
23. Milner J, Gonçalves F, Gonçalves L. A pacemaker pocket mass has many faces. J Cardiol Cases. 2021 May 15;24(5):244-246. doi: 10.1016/j.jccase.2021.04.005. PMID: 34868407; PMCID: PMC8617474.
24. Moruzzo D, Bindi M, Bongiorni MG, Castiglioni M. A rare case of non-Hodgkin lymphoma in a pacemaker pocket. Leuk Lymphoma. 2009 Aug;50(8):1384-5. doi: 10.1080/10428190903039990. PMID: 19562613.
25. Nemec J, Swerdlow SH, Bazaz R, Saba SF, Shalaby AA. B-Cell lymphoproliferative disorder of an ICD pocket: a diagnostic puzzle in an immunosuppressed patient. Pacing Clin Electrophysiol. 2008 Jun;31(6):769-71. doi: 10.1111/j.1540-8159.2008.01084.x. PMID: 18507553.
26. Hojo N, Yakushijin Y, Narumi H, Minamoto Y, Sakai I, Takada K, Hato T, Yasukawa M, Fujita S. Non-Hodgkin's lymphoma developing in a pacemaker pocket. Int J Hematol. 2003 May;77(4):387-90. doi: 10.1007/BF02982649. PMID: 12774929.
27. Patris V, Argiriou M, Lama N, Constantinou P, Charitos C. Primary B-cell lymphoma developing at epicardial pacemaker lead site. J Card Surg. 2014 Sep;29(5):763. doi: 10.1111/jocs.12403. Epub 2014 Jul 16. Erratum in: J Card Surg. 2015 May;30(5):476. Haq, Iram [removed]. PMID: 25041447.
28. Perucki WH, Makkapati S, Laslett DB, Cooper JM. Device infection mimicry: Physical examination characteristics and procedural technique for pacemaker-associated skin cancer. HeartRhythm Case Rep. 2023 Jul 20;9(10):698-700. doi: 10.1016/j.hrcr.2023.07.007. PMID: 38047204; PMCID: PMC10691937.
29. Popelier B, Vanheste R, Cuypers S, Heggermont W. An unexpected cause of a swollen pacemaker pocket: a case report. Eur Heart J Case Rep. 2022 May 24;6(6):ytac211. doi: 10.1093/ehjcr/ytac211. PMID: 35685031; PMCID: PMC9174550.
30. Li Q. Mass in a pacemaker pocket. Kardiol Pol. 2017;75(9):933. doi: 10.5603/KP.2017.0171. PMID: 28895992.
31. Rasmussen K, Grimsgaard C, Vik-Mo H, Stalsberg H. Male breast cancer from pacemaker pocket. Pacing Clin Electrophysiol. 1985 Sep;8(5):761-3. doi: 10.1111/j.1540-8159.1985.tb05891.x. PMID: 2414760.
32. Rathinam S, Kuntz H, Panting J, Kalkat MS. Inflammatory myofibroblastic tumour at the pacemaker site. Interact Cardiovasc Thorac Surg. 2010 Mar;10(3):443-5. doi: 10.1510/icvts.2009.221945. Epub 2009 Dec 29. PMID: 20040480.
33. Reyes CV. Clear cell hidradenocarcinoma developing in pacemaker pocket. Pacing Clin Electrophysiol. 2008 Nov;31(11):1513-5. doi: 10.1111/j.1540-8159.2008.01217.x. PMID: 18950311.
34. Ribero S, Pullara A, Caliendo V, Lauro D, Balagna EM, Cristoforetti Y, Golzio PG, Gaita F, Quaglino P, Bernengo MG, Macripò G. Melanoma relapse over a pacemaker pocket: case report and integrated management strategies. Minerva Chir. 2013 Jun;68(3):329-32. PMID: 23774099.
35. Rothenberger-Janzen K, Flueckiger A, Bigler R. Carcinoma of the breast and pacemaker generators. Pacing Clin Electrophysiol. 1998 Apr;21(4 Pt 1):769-71. doi: 10.1111/j.1540-8159.1998.tb00137.x. PMID: 9584311.
36. Sasaki M, Katoh Y, Niwakawa M, Andoh A, Hodohara K, Fujiyama Y, Bamba T, Hosoda S, Kanoh T. [Multiple myeloma with a mass formation in a pacemaker pocket]. Rinsho Ketsueki. 1992 Nov;33(11):1747-52. Japanese. PMID: 1469792.
37. Cutaneous Plasmacytoma: A Rare Complication of Multiple Myeloma and Permanent Pacemaker Insertion Selvarajah, A. et al. Heart, Lung and Circulation, Volume 27, S159
38. Snorek M, Bulava A, Vonke I. Chronic lymphocytic leukemia skin infiltration mimicking an ICD pocket infection: a case report. BMC Cardiovasc Disord. 2017 Mar 24;17(1):89. doi: 10.1186/s12872-017-0522-5. PMID: 28340568; PMCID: PMC5364587.
39. Tada H, Asazuma K, Naiki H, Nakai T, Nakakuki K. Metastatic tumor thrombus attached to a pacemaker electrode. Pacing Clin Electrophysiol. 1998 Nov;21(11 Pt 1):2143-6. doi: 10.1111/j.1540-8159.1998.tb01137.x. PMID: 9826870.
40. Tanaka K, Ohyama K, Tomita K, Sawada K, Kawara T, Kosuga K, Aoyagi S. [Breast cancer at the site of an implanted pacemaker]. Kyobu Geka. 1999 Jun;52(6):496-9. Japanese. PMID: 10380480.
41. Yong AA, Stefanato CM, Attard NR, Mackenzie Ross A, Mallipeddi R. An ulcerated nodule over an implanted cardiac defibrillator: A journey from presumed infection to leiomyosarcoma. Australas J Dermatol. 2017 Aug;58(3):e148-e149. doi: 10.1111/ajd.12524. Epub 2017 Jun 30. PMID: 28664552.
42. Zarifi C, Deutsch S, Dullet N, Mukherjee KK, Mukherjee A, Abubaker F. An enlarging pacemaker pocket: A case report of a plasmablastic lymphoma arising as a primary tumor around a cardiac pacemaker and systematic literature review of various malignancies arising at the pacemaker pocket. J Cardiol Cases. 2017 Oct 13;17(2):41-43. doi: 10.1016/j.jccase.2017.09.006. PMID: 30279851; PMCID: PMC6149566.
43. Zonca P, Herokova J, Cambal M, Jacobi CA. Ductal carcinoma of the breast in the pacemaker generator's pocket. Bratisl Lek Listy. 2009;110(11):719-22. PMID: 20120443.
44. Adamo F, Colaiaco C, Mahfouz K, Finamora I, Danisi N, Pappalardo A, Vergoni F, Ammirati F, Santini L. Anaplastic Large Cell Lymphoma Growing in the Pacemaker Pocket. JACC Case Rep. 2024 Aug 7;29(15):102415. doi: 10.1016/j.jaccas.2024.102415. PMID: 39157575; PMCID: PMC11328755.
45. Agnieszka Nowosielecka, Dorota Nowosielecka, Kamil Karpeta, Janusz Gozdek, Andrzej Kutarski.Pocket hematoma that turned out to be cancer. Oncology in Clinical Practice. 2024. Doi: 10.5603/ocp.100238.
