# Supplementary material for: Neoplasms arising at the CIED pocket: a hybrid study combining a case report, scoping review, and clinical survey
Source: Intern Emerg Med. 2026 Jan 8;21(3):957–64. doi: 10.1007/s11739-025-04251-4 (PMC13144183; doi:10.1007/s11739-025-04251-4)
Supplement: Supplementary file 2 — Supplementary file2 (DOCX 18 KB) [file 11739_2025_4251_MOESM2_ESM.docx]

**Supplementary Table 2. Quality assessment for case series.**

|  | **JBI Critical Appraisal Checklist for Case Series** | | | | | | | | | |
| --- | --- | --- | --- | --- | --- | --- | --- | --- | --- | --- |
| Ref. | 1 | 2 | 3 | 4 | 5 | 6 | 7 | 8 | 9 | 10 |
|  | Y | Y | Y | N | N | Y | N | N | N | NA |
|  | Y | Y | Y | N | N | Y | Y | N | N | NA |
|  | Y | Y | Y | N | N | N | Y | Y | Y | NA |
|  | Y | Y | Y | N | N | Y | Y | Y | Y | NA |
|  | Y | Y | Y | N | N | N | N | Y | Y | NA |

1. Were there clear criteria for inclusion in the case series?
2. Was the condition measured in a standard, reliable way for all participants included in the case series?
3. Were valid methods used for identification of the condition for all participants included in the case series?
4. Did the case series have consecutive inclusion of participants?
5. Did the case series have complete inclusion of participants?
6. Was there clear reporting of the demographics of the participants in the study?
7. Was there clear reporting of clinical information of the participants?
8. Were the outcomes or follow-up results of cases clearly reported?
9. Was there clear reporting of the presenting sites’/clinics’ demographic information?
10. Was statistical analysis appropriate?

Yes: Y. No: N. Unclear: U. Not Applicable: N.A.

Moola S, Munn Z, Tufanaru C, Aromataris E, Sears K, Sfetcu R, Currie M, Lisy K, Qureshi R, Mattis P, Mu P. Chapter 7: Systematic reviews of etiology and risk. In: Aromataris E, Munn Z (Editors)*. JBI Manual for Evidence Synthesis.* JBI, 2020. Available from https://synthesismanual.jbi.global. https://doi.org/10.46658/JBIMES-20-08

**Reference**

1. Bhandarkar DS, Bewu AD, Taylor TV. Carcinoma of the breast at the site of migrated pacemaker generators. Postgrad Med J. 1993 Nov;69(817):883-5. doi: 10.1136/pgmj.69.817.883. PMID: 8290439; PMCID: PMC2399934.
2. Biran S, Keren A, Farkas T, Stern S. Development of carcinoma of the breast at the site of an implanted pacemaker in two patients. J Surg Oncol. 1979;11(1):7-11. doi: 10.1002/jso.2930110103. PMID: 219300.
3. Dalal JJ, Wingerbottam T, West RR, Henderson AH. Implanted pacemakers and breast cancer. Lancet. 1980 Aug 9;2(8189):311. doi: 10.1016/s0140-6736(80)90253-6. PMID: 6105454.
4. Moseley T, Birgersdotter-Green U, Feld G, Pollema T. Malignancies masquerading as device pocket infections. HeartRhythm Case Rep. 2021 Jul 31;7(10):694-697. doi: 10.1016/j.hrcr.2021.07.009. PMID: 34712568; PMCID: PMC8530908.
5. Zafiracopoulos P, Rouskas A. Letter: Breast cancer at site of implantation of pacemaker generator. Lancet. 1974 Jun 1;1(7866):1114. doi: 10.1016/s0140-6736(74)90596-0. PMID: 4135554.
