# Supplementary figures and images for: Neoplasms arising at the CIED pocket: a hybrid study combining a case report, scoping review, and clinical survey
Source: Intern Emerg Med. 2026 Jan 8;21(3):957–64. doi: 10.1007/s11739-025-04251-4 (PMC13144183; doi:10.1007/s11739-025-04251-4)

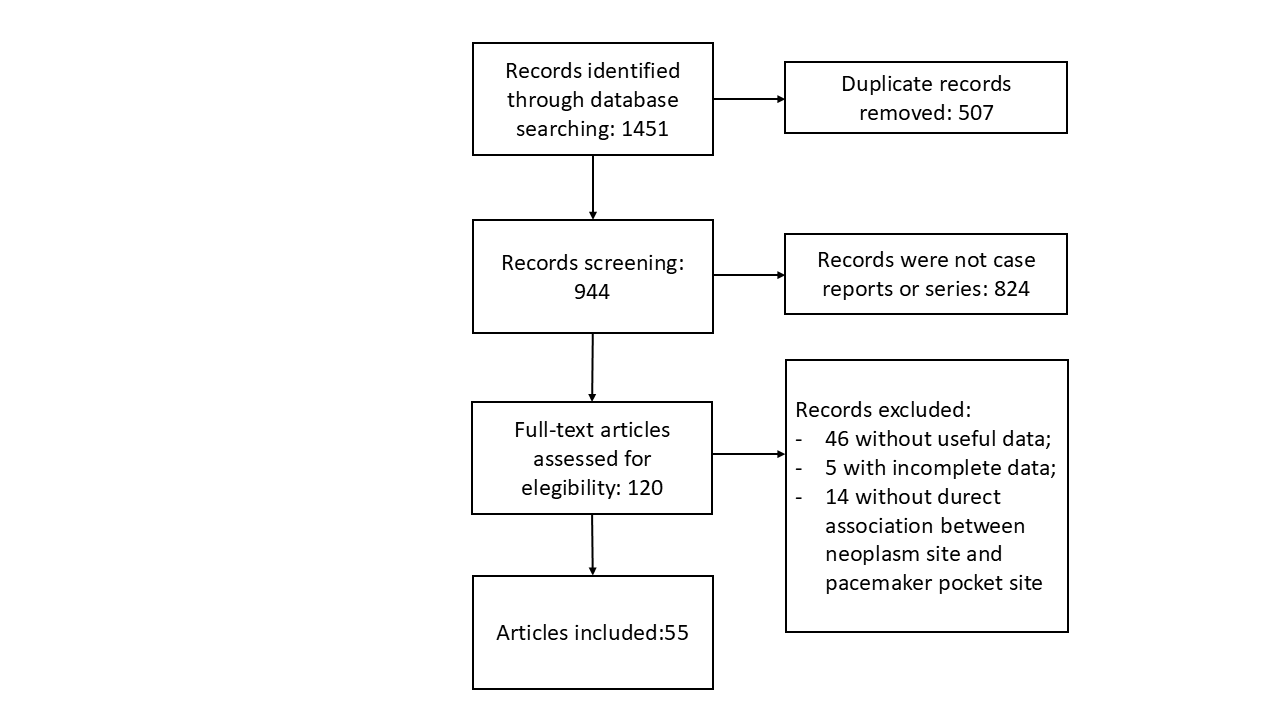

Supplement: Supplementary file 3 — Supplementary file3 (TIF 96 KB) [file 11739_2025_4251_MOESM3_ESM.tif]
